# Intestinal goblet cell carcinoid presenting with recurrent sterile peritonitis in a patient on peritoneal dialysis: a case report

**DOI:** 10.1186/s12882-017-0477-x

**Published:** 2017-02-13

**Authors:** Chih-Wei Chen, Jan-Show Chu, Li-Chun Hsieh, Chih-Chin Kao, Yen-Chung Lin, Hsi-Hsien Chen

**Affiliations:** 1 Division of Nephrology, Department of Internal Medicine, Taipei Medical University Hospital, Taipei, Taiwan; 2 Department of Pathology, School of Medicine, College of Medicine, Taipei Medical University, Taipei, Taiwan; 3 Department of Medical Imaging, Taipei Medical University Hospital, Taipei, Taiwan; 4 Translational Imaging Research Center, College of Medicine, Taipei Medical University, Taipei, Taiwan; 5 Division of Nephrology, Department of Internal Medicine, School of Medicine, College of Medicine, Taipei Medical University, Taipei, Taiwan

**Keywords:** Case report, Goblet cell carcinoid, Sterile peritonitis, Peritoneal dialysis

## Abstract

**Background:**

Goblet cell carcinoid is a rare variant of appendiceal carcinoid with mixed endocrine and exocrine features. The most common symptom and signs are abdominal pain, acute appendicitis and palpable mass. Additionally, abdominal pain is common in patient on peritoneal dialysis, which may confound the diagnosis in such patient.

**Case presentation:**

We report a 71- years- old woman on peritoneal dialysis that experienced several episodes of abdominal cramping pain and sterile peritonitis. She had one episode of severe pain and underwent an appendectomy for suspicion of appendicitis. Goblet cell carcinoid was diagnosed. She had no further abdominal pain after she received appendectomy.

**Conclusions:**

Malignant dialysate was rarely reported in patient with peritoneal dialysis. However, goblet cell carcinoid can initially present with acute appendicitis, chronic intermittent abdominal pain and mimicking peritonitis. In systemically reviewing the literature, this is the first case report of sterile peritonitis with peritoneal dialysis caused by goblet cell carcinoid.

## Background

Goblet cell carcinoid is a rare variant of appendiceal carcinoid with mixed endocrine and exocrine features. The most common clinical presentation is acute appendicitis [1, 2]. Typically, GCC tumors are infiltrative and involve the entire appendix circumferentially [3]. Nevertheless, Tang et al. [1] reported that abdominal pain and palpable mass are also reported presented in 50% of GCC patients. Additionally, abdominal pain is common in patients on peritoneal dialysis, which may confound the diagnosis in such patient. For these patients, right hemicolectomy has been traditionally suggested by physician [2]. We performed systematic review of PubMed and Cochrane Library databases and noted no similar cases of goblet cell carcinoid causing abdominal pain which mimics peritoneal dialysis related peritonitis. We report this unique case of GCC presenting with sterile peritonitis in a patient on peritoneal dialysis to prompt clinicians to consider GCC in the differential diagnosis of similar symptoms in such patients.

## Case presentation

**Fig. 1:**
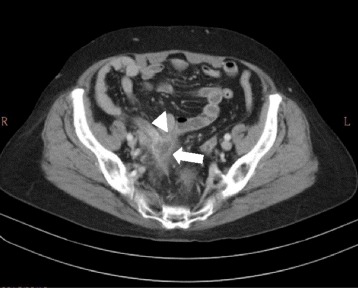
Contrast-enhanced pelvic computed tomography. The scan reveals one 2.1 × 1.6 cm irregular-shaped cystic mass (*arrow*) with marginal enhancement in the right pelvic cavity, abutting the swollen appendix (*arrowhead*). The tumor is located at the tip of appendix with possible subserosa invasion and attaches to the right pelvic side wall. The scan also shows inflammatory change with regional ileal bowel loop adhesion to the mass. Some fluid collection around the tumor-bowel complex is noted

**Fig. 2:**
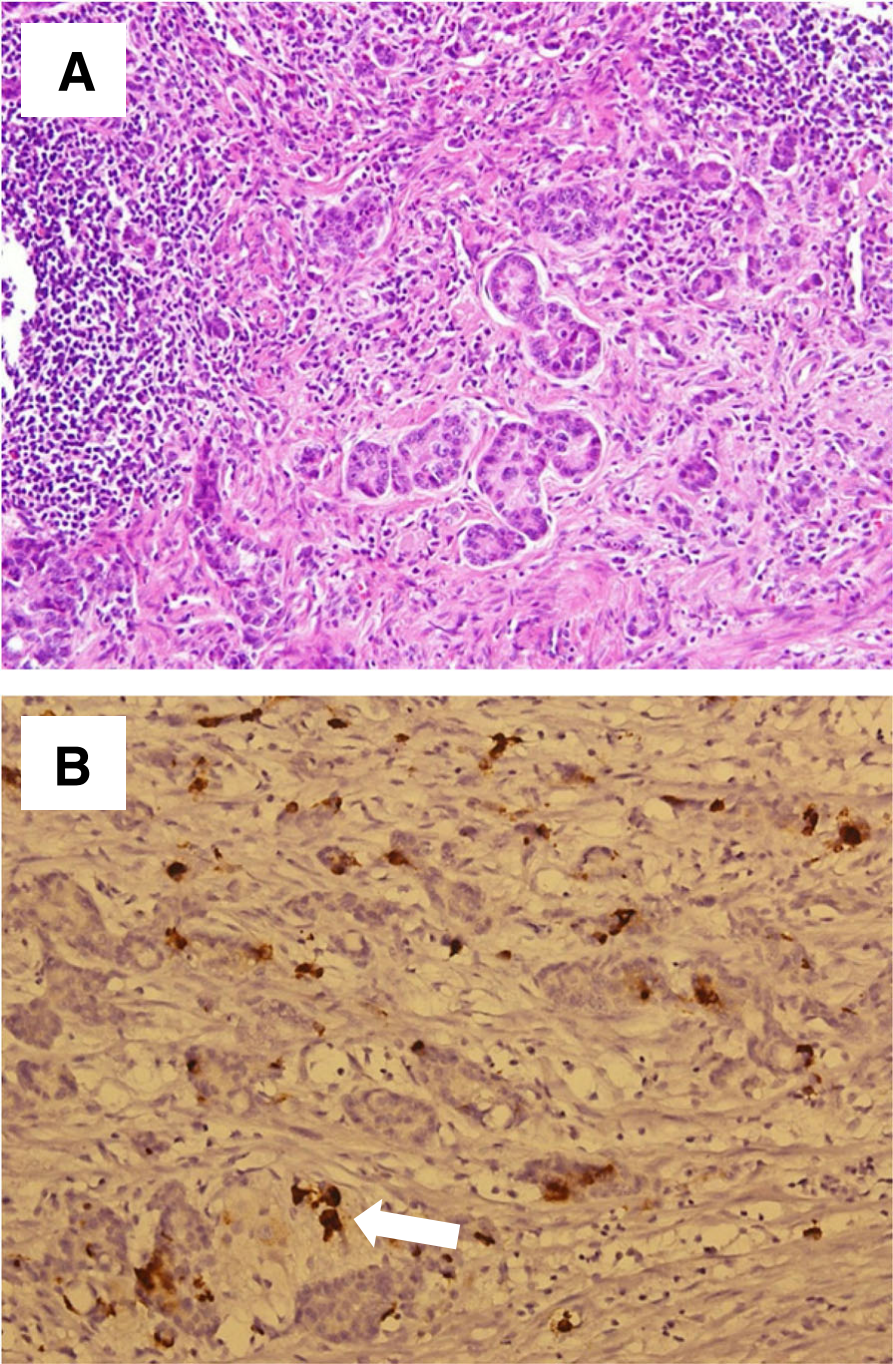
Peritoneum obtained by laparotomy. (200X) **a** H & E staining shows typical goblet cell carcinoid. The photomicrography shows goblet cell carcinoid composed of concentric infiltrating neoplastic cells in the appendiceal wall, including the mucosa, submucosa, muscular propria, and subserosal layers. The infiltrated wall shows fibrotic change. The tumor includes small solid or tubule-like tumor clusters, displaying monotonous nuclei with fine chromatin and eosinophilic cytoplasm, and occasional mucin-producing goblet cells arranged singly or small nests. **b** The Immunohistochemical study is positive for chromogranin A Brown color (*arrow*)

A 71- years- old woman with underlying hypertension and end- stage renal disease on peritoneal dialysis, experienced several episodes of abdominal cramping pain with mild diarrhea 3 months after starting peritoneal dialysis. She had chills but no fever. A physical examination showed lower abdominal tenderness, but no rebounding pain. Initial complete blood count showed no leukocytosis (8400/μL). However, the initial dialysate studies showed increasing leukocytes with a predominance of neutrophils in first few sample, shifting to lymphocyte predominance in later sample (Table 1). Dialysate and blood cultures from 15 sets over 4 months since initial abdominal pain showed no growth. Since empirical antibiotic treatment did not improve the patient’s condition of recurrent cramping pain, abdominal computerized tomography (CT) was performed which revealed no abnormality. The patient still complained intermittent abdominal pain after the CT, so pain control medication (including acetaminophen and tramadol) was prescribed for the patient. The Tenckhoff catheter was removed 2 months after the onset of abdominal pain, and then hemodialysis was started. Laparotomy and biopsy of peritoneal were performed during removal of Tenckoff catheter. Staining for acid-fast bacilli was negative. She continued to complain of intermittent abdominal cramping pain. One and half year after her initial abdominal pain, abdominal CT was again performed due to an episode of particularly severe abdominal pain. The scan showed fluid collection in the right pelvic cavity extending to the distal part of the appendix (Fig. 1). An appendectomy was immediately performed, and goblet cell carcinoid (GCC) with suppurative appendicitis was diagnosed. The CT scan before patient received appendectomy show the swollen appendix without obvious extraappendiceal lesion. On the other hand, according to the surgical and histopathological findings, the appendix was perforated with adhesion to ileum, accompanied by regional inflammatory changes in pelvic cavity. Microscopically, the infiltrating neoplastic cells were found in the appendiceal wall, including mucosa, submucosal, muscular propria, and subserosal layers. The infiltrated wall also showed fibrotic change. No evidence extra-appendiceal invasion of the tumor could be found (Fig. 2, the pathology specimen of this patient). Right hemicolectomy and adjuvant chemotherapy were suggested for complete treatment of GCC but the suggestions were refused by the patient. No further abdominal pain has been noted since appendectomy, and the patient still visits oncologist outpatient department for regular follow up without evident of recurrent tumor.

**Table 1:** Serial dialysate analysis of patient during episodes of abdominal pain in 2012

Date	09/13	09/16	09/19	09/21	09/25	10/02	10/12	11/19
Specific gravity	1.010	1.018	1.010	1.010	1.014	1.014	1.010	1.010
Red blood cells/mm^3^	6	180	63	297	126	81	75	9
White blood cells/mm^3^	136	648	243	90	144	222	252	494
Neutrophil:Lymphocyte:H:M	24:21:48:2:5(E)	80:20	48:16:28:8	24:27:48:1	35:32:22:4:7	10:81:1:8(E)	4:92:4	44:53:1:2

*H* macrophage, *M* mesothelial, *E* Eosinophil

## Discussion and Conclusions

Peritonitis in during peritoneal dialysis is a significant complication in clinically which can cause death or structural changes of the peritoneum [4]. Peritoneal infection is also a common cause of dialysis patients switching the therapy from peritoneal dialysis to hemodialysis [5]. On the other hand, sterile peritonitis is a common issue in the patients receiving peritoneal dialysis. Sterile peritonitis, culture-negative peritonitis, is often caused by antecedent empirical antibiotic treatment before dialysate culture are collected for culture. Additionally, infection caused by atypical organisms or non-infectious causes, can also lead to sterile peritonitis [6]. Peritoneal dialysate cultures prove to be negative in up to 22% of case [6]. Patients using icodextrin for peritoneal dialysis often develop peritonitis with an associated non-neutrophil predominant increasing in dialysate white blood cells [7]. Tuberculous (TB) peritonitis and malignancy related peritonitis also cause similar changes [6]. However, non-neutrophil-dominant increases in dialysate white blood cells in association with malignancy dialysate are rare [8, 9]. Lymphoma sometimes mimics peritonitis in patients on ambulatory peritoneal dialysis [8]. Peritoneal metastases can also mimic peritonitis [9].

Goblet cell carcinoid (GCC), an unique and enigmatic tumor involving the appendix almost exclusively, is a rare variant of appendiceal carcinoid [10]. Carcinoid tumors account for up to 85% of all appendiceal tumors, white, GCC accounts for only 6% of all appendiceal carcinoid tumors [11]. The term GCC was firstly introduced by Subbuswamy et al. in 1974 [12]. GCC derive from a pluripotent intestinal stem cell that differentiates into both mucinous and neuroendocrine cells [13]. Thus, it shares histologic features of both adenocarcinomas and carcinoid tumors. Additionally, GCC is reportedly more aggressive and unpredictable in nature than other carcinoid tumors.

The most common clinical presentation is acute appendicitis [1]. Abdominal pain and a palpable mass are also reported by 50% of GCC patients [2]. Pham et al. [1]. also reported other symptoms including bowel obstruction, intussusception, gastrointestinal bleeding, and chronic intermittent lower abdominal pain. Compared with other carcinoid tumors which are usually asymptomatic, GCC often presents with clinical symptoms. Thus, the incidental diagnostic rate is only about 3% [2]. Despite serotonin can be detected immunohistochemically in GCC tumor [14], there is no carcinoid syndrome has been reported in GCC patients and urinary 5-hydroxyindoleacetic acid (5-HIAA) levels in these patients are usually within normal limits, as opposed to carcinoid tumors, which are commonly with elevated urinary 5-HIAA and serum chromogranin A [15].

The most common route of metastases is trans-coelomic/peritoneal invasion, and the most common sites involved are ovaries, and the peritoneal surfaces of the pelvis and abdominal cavity [10].

The natural history of patients with GCC is intermediate in aggressiveness between adenocarcinomas and classical carcinoids [10]. Based on its natural history and malignant nature, optimal therapeutic startegies are in general similar to adenocarcinomas rather than classical carcinoids [10]. Thus, surgical management with right hemicolectomy is recommended after appendectomy for most cases, particularly those with an adenocarcinoma components [2]. Nevertheless, right hemicolectomy is a significant abdominal procedure with an associated risk, especially in the infirm or elderly [15]. Peritoneal carcinomatosis is the most common cause of death [10]. Goblet cell carcinooids are associated with a 60% 10-year survival and is similar to survival after treatment of a low-risk adenocarcinoma [15]. The prognosis of GCC is intermediate between appendiceal carcinoids and adenocarcinomas [10].

Our patient, who experienced abdominal pain intermittently for months had been diagnosed with peritonitis related to peritoneal dialysis. Given a clinical history of recurrent sterile peritonitis with a predominance of lymphocytes in the dialysate, an atypical infection (including tuberculosis) and intra-abdominal malignancy must be considered in the differential diagnosis. However, no dialysate or ascites was sent to cytology analysis in our patient. We also considered intermittent bowel obstruction as the cause of abdominal pain, which was the common presentation of GCC [1], but there were no sufficient evidence to support the possibility.

Of all the carcinoid tumors, GCC, in particular, can initially present with acute appendicitis, chronic intermittent abdominal pain, and malignant ascites mimicking peritonitis related to peritoneal dialysis in such patient. Through systematic review of PubMed and Cochrane Library databases, there were no similar cases reported. We report this unique case of GCC presenting with sterile peritonitis in a patient on peritoneal dialysis to prompt clinicians to consider GCC in the differential diagnosis of similar symptoms in such patients.

## Abbreviations

5-HIAA: 5-hydroxyindoleacetic acid
CT: Computerized tomography
GCC: Goblet cell carcinoid
